# Ontario Veterinary Medical College Society

**Published:** 1896-11

**Authors:** C. H. Sweetapple


Secretary.

### ONTARIO VETERINARY MEDICAL COLLEGE SOCIETY.

A MEETING of this Society was held on Wednesday evening, October 14th, for the purpose of electing officers for the academic year, with the following result: Permanent officers: Andrew Smith, F.R.C.V.S., President; James Thorburn, M.D., First Vice-President; J. T. Duncan, M.D., V.S., Second Vice-President; and C. H. Sweetapple, V.S., Third Vice-President. Elective officers: W. W. Richards, San Diego, Cal., Secretary; R. C. Cliff, Hamilton, Ontario, Assistant Secretary; A. C. Tweedie, Carson City, Mich., Treasurer; and G. C. Bowen, Newark, N. J., Librarian.

The Society meets bi-weekly for the reading and discussion of papers and essays on professional subjects, and it is our object to have interesting and instructive meetings.

The Society held its first meeting of the session 1896–97 for the reading and discussion of papers in the lecture hall of the College, on Friday evening, October 16th. Prof. Smith, F.R.C.S.V., Principal, presided. There was a good attendance of students, who, as usual, were assembled here from Canada and our great Northwest—all parts of the United States, Jamaica, and also Great Britain being represented.

The chairman, in opening the meeting, made a few well-chosen remarks announcing the objects of the meetings and the benefits to be derived from a free discussion and interchange of views on the papers submitted. The following papers were read: "Crotalism," a disease affecting the horse, produced by eating the plant called the Rattle Box (*Crotalyinna Sagittatis*), which possesses narcotic qualities, by Mr. G. P. Statter; "Laminitis," by Mr. C. H. Bugbee; "Azoturia," by Mr. T. J. Cooper; "Inversion of the Uterus," by Mr. C. W. Clark.

The papers were well received and were listened to with marked attention, and at the close of each paper very valuable and instructive discussions ensued. C. H. SWEETAPPLE.

